# Circulating activated immune cells as a potential blood biomarkers of non-small cell lung cancer occurrence and progression

**DOI:** 10.1186/s12890-021-01636-x

**Published:** 2021-09-06

**Authors:** Yingyi Wang, Na Zhou, Rui Zhu, Xiaoyuan Li, Zhao Sun, Yang Gao, Wei Liu, Changting Meng, Yuping Ge, Chunmei Bai, Taisheng Li, Hongsheng Liu

**Affiliations:** 1 Department of Medical Oncology, Peking Union Medical College Hospital, Chinese Academy of Medical Sciences and Peking Union Medical College, Beijing, China; 2 Department of Medical Record, Peking Union Medical College Hospital, Chinese Academy of Medical Sciences and Peking Union Medical College, Beijing, China; 3 Department of Intervention Group of Radiology, Peking Union Medical College Hospital, Chinese Academy of Medical Sciences and Peking Union Medical College, Beijing, China; 4 Institute for Systems Biology, Seattle University, Seattle, WA 98109 USA; 5 Department of Infectious Diseases, Peking Union Medical College Hospital, Chinese Academy of Medical Sciences and Peking Union Medical College, No. 1 Shuaifuyuan Wangfujing Dongcheng District, Beijing, 100730 China; 6 Department of Thoracic Surgery, Peking Union Medical College Hospital, Chinese Academy of Medical Sciences and Peking Union Medical College, No. 1 Shuaifuyuan Wangfujing Dongcheng District, Beijing, 100730 China

**Keywords:** Immune cells, NSCLC, Cancer occurrence, Advance cancer stage, Clinicopathologic characteristics

## Abstract

**Background:**

Treatment for non-small cell lung cancer (NSCLC) has greatly improved in recent years. However, noninvasive early screening for carcinogenesis and progression unclear. The aim of this study was to explore the predictive value of peripheral blood immune cells in untreated NSCLC patients.

**Methods:**

We retrospectively enrolled 305 untreated NSCLC patients and 132 healthy participants from February 2016 to August 2019 in Peking Union Medical College Hospital. Immune cell levels were determined by flow cytometry and routine blood tests.

**Results:**

NSCLC patients had lower levels of T lymphocytes, NK cells, CD8+ T cells, naïve CD4+/CD4+, naïve CD4+ T cells and higher levels of CD4+ T cells, memory CD4+/CD4+ T cells, memory CD4+ T cells, CD4+CD28+/CD4+ T cells, CD4+CD28+ T cells, CD8+CD28+/CD8+ T cells, CD8+HLA-DR+/CD8+ T cells, CD8+HLA-DR+ T cells T cells, CD8+CD38+/CD8+ T cells, CD8+CD38+ T cells and CD4+/CD8+ T cells than those in controls. The percentages of specific lymphocyte subtypes were significantly different in cancer patients versus healthy individuals. For instance, cancer patients had lower levels of B cells, CD4+ T cells, naïve CD4+/CD4+ T cells, naïve CD4+ T cells, CD4+CD28+ T cells, CD8+CD28+ T cells and higher levels of NK cells, white blood cells (WBC), monocytes, neutrophils, eosinophils, basophils, monocytes to lymphocyte ratio (MLR), neutrophils to lymphocyte ratio (NLR), eosinophil to lymphocyte ratio (ELR), basophil to lymphocyte ratio (BLR), and blood platelet to lymphocyte ratio (PLR).

**Conclusions:**

Abnormal T cell levels can be used as an independent predictive biomarker for noninvasive early screening in NSCLC occurrence and progression.

**Supplementary Information:**

The online version contains supplementary material available at 10.1186/s12890-021-01636-x.

## Background

Lung cancer is the leading cause of cancer-related disease incidence and mortality worldwide (11.6% and 18.4% of the total cases, respectively) [1, 2]. NSCLC accounts for approximately 80–85% of lung cancers with a 5-year survival rate of less than 15% for advanced cancer [3, 4]. The 5-year survival ranges from 50 to 80% for early stage NSCLC treated with surgical resection. However, the diagnosis of early-stage NSCLC occurs in less than 20% of cases [5]. Improving the accuracy of prediction could contribute to enabling a better treatment strategy [6]. Thus, it is important to identify markers to predict the advanced cancer stage of patients with lung cancer upon noninvasive method.

In recent years, the role of the immune system has been an increasingly recognized in cancer development and progression. Immune cells play critical roles in the anti-tumor response basing on promoting or suppressing tumor progression and subsequent invasion and metastasis [7]. To identify new predictive markers, tumor infiltrating T-lymphocytes have become a hot topic of research and several researches have demonstrated their predictive role in cancer [8]. However, the detection of TILs is complex and cannot be dynamically monitored. In this context, there has been a great focus on peripheral blood, which is the main source of immune cells, which has several advantages including simpler handling, noninvasive, and the possibility of dynamic monitoring.

Several studies have reported the levels and roles of peripheral blood lymphocyte subsets in NSCLC, such as B cells, CD4+ T cells, and CD4/CD8+ T cell ratio [9, 10]. The relationships between lymphocyte subsets and gender, age and stage were also reported [11]. However, the predictive values of immune cells in untreated lung cancer patients have not been well studied. In this study, we analyzed peripheral blood immune cells to provide basic data for further exploration of tumor predictive indicators.

## Methods

### Patients and clinical data

A total of 437 participants were recruited atPeking Union Medical College Hospital (PUMCH) between February 2016 and August 2019 and had not received anti-tumor therapies before enrollment. 305 untreated NSCLC patients (141 male and 164 female) were selected with ages between 25 and 84 years (mean age: 59.67 years). 135 patients had no active disease with surgery before diagnosed lung cancer and 43 patients had received two surgeries. 211 patients had conformed history of diseases before being diagnosed with lung lung cancer including 142 patients who suffered from two diseases. 84 patients had smoking history with 1 to 63 years including 51 patients with a smoking cessation from 0.1 to 30 years. 67 patients had a drinking history including abstinence for 10 patients. 132 age- and sex-matched healthy volunteers (96 men and 53 women) were selected with age from 25 to 80 years (mean age: 59.19 years). Age was divided into three groups upon World Health Organization (Yong: 0–44 years; Middle people: 45–59 years; Elderly people: over 59 year). The clinical data of untreated patients are summarized in Table 1. All participants gave informed consent. This study was approved by the Ethical Committee of PUMCH (JS-1405).

**Table 1:** Clinicophthological characterstics of the untreated lung cancer patients in this study

Characterstics	N = 305
Gender	
Male	141
Female	164
Age	
Yong	25
Middle	116
Elder	164
Allergic history	
Antibiotic	31
Other	6
No allergic	239
Unkown	29
Surgery	
Uterine	27
Caesarean section	11
Epityphlon	24
Thyroid	17
Intestines	13
Other	86
No surgery	146
Unkown	24
History of diseases	
Hypertension	110
Diabetes	40
Coronary heart disease	21
Thyroid nodule	19
Fatty liver	11
Other	152
No Medical	75
Unkown	19
Smoking history	
Yes	33
Cessation	51
No	193
Unkown	19
Drinking history	
Yes	57
Abstinence	10
No	219
Unkown	19
ECOG PS	
0	247
1	35
2	9
3	2
Unkown	12
Histology	
Adenocarcinoma	277
Squamous carcinoma	27
Adenosquamous carcinoma	1
Stage	
I	203
II	18
III	27
IV	46
Unkown	11
Tumour stage	
T1	207
T2	42
T3	16
T4	22
Unkown	18
Lymph nodes metastases	
N0	215
N1	12
N2	33
N3	24
Unkown	21
Distant metastases	
M0	243
M1	46
Unkown	16

### Flow cytometry and blood routine tests

Lymphocyte immunophenotyping was conducted by three-color flow cytometry (Epics XL flow cytometry; Beckman Coulter, USA). Specific monoclonal antibodies against CD19, CD16CD56, CD4, CD8, CD45RO, CD45RA, CD28, HLA-DR, and CD38 were used to identify lymphocyte subsets. A dual-platform method was performed to calculate lymphocyte subsets upon WBC counts. Inflammatory cells including lymphocytes, monocytes, neutrophils, eosinophils, basophils, red blood cells (RBC), hemoglobin, platelet were acquired from routine blood tests of the same sample. In addition, the levels of MLR, NLR, ELR, BLR, red blood cells to lymphocyte ratio (RLR), hemoglobin to lymphocyte ratio (HLR), and PLR were evaluated.

### Statistical analysis

Statistical analysis was performed using SPSS 22.0 software (IBM Corporation, USA) and GraphPad Prism 7.0 software (San Diego, USA). The data were expressed using means ± standard deviation. Kolmogorov–Smirnov test was performed for the distribution test. Normally distributed were analyzed by t-test and one-way analysis. Non-parametric data were compared by Mann–Whitney test and Kruskal–Wallis. Spearman’s rank correlation test was used for correlation analysis. Probability value was performed 2-sided tests and *p* < 0.05 was considered statistically significant.

## Results

### Comparison of immune parameters in NSCLC versus healthy individuals

To explore the predictive role of immune cells in untreated NSCLC patients, a total of 487 Chinese adults (305 lung cancer patients and 132 healthy controls) were enrolled in this study. We did not analyze inflammatory cells due to a lack of these data for controls.The levels of lymphocyte subsets were significantly associated with gender and age in healthy controls and cancer patients, thus we carefully avoided age- and sex-related biases.

We compared the levels of immune cells in all patients and controls based on t-test and Mann–Whitney test. In this study, low levels of T lymphocytes (*p*** < 0.001**), NK cells (*p*** < 0.001**), CD8+ T cells (*p* = 0.008), naïve CD4+/CD4+ (*p*** < 0.001**), and naïve CD4+ T cells (*p*** < 0.001**) was observed in lung cancer patients compared to controls. However, levels of CD4+ T cells (*p*** = 0.042**), memory CD4+/CD4+ (*p*** < 0.001**), memory CD4+ T cells (*p*** < 0.001**), CD4+CD28+/CD4+(*p*** < 0.001**), CD4+CD28+ T cells (*p*** = 0.002**), CD8+CD28+/CD8+ (*p*** = 0.004**), CD8+HLA-DR+/CD8+ (*p*** < 0.001**), CD8+HLA-DR+ T cells (*p*** = 0.022**), CD8+CD38+/CD8+ (*p*** < 0.001**), CD8+CD38+ T cells (*p*** = 0.001**) and CD4+/CD8+(*p*** < 0.001**) were higher in patients than those in controls. There was no significant difference for B cells and CD8+CD28+ T cell counts between patients and controls (p > 0.05). The result was shown in Table 2.

**Table 2:** Differences of immune parameters in untreated lung cancer patients and healthy controls

Lymphocyte subsets	Healthy controls (N = 132)	Lung cancer patients (N = 357)	P value
T Lymphocyte (cells/10^12ul)	1.97 ± 0.53	1.73 ± 0.61	** < 0.001**
B cells (cells/ul)	201.69 ± 91.56	184.85 ± 98.29	0.054
NK cells (cells/ul)	390.99 ± 251.48	269.15 ± 213.78	** < 0.001**
CD4+ T cells (cells/ul)	689.83 ± 255.28	745 ± 302.8	**0.042**
CD8+ T cells (cells/ul)	511.43 ± 255.09	439.25 ± 212.9	**0.008**
Memory CD4+/CD4+ (%)	65.89 ± 13.8	73.55 ± 12.88	** < 0.001**
Memory CD4+ T cells (cells/ul)	441.8 ± 166.31	539.09 ± 220.41	** < 0.001**
Naïve CD4+/CD4+ (%)	34.11 ± 13.8	24.21 ± 12.2	** < 0.001**
Naïve CD4+ T cells (cells/ul)	248.05 ± 160.27	183.7 ± 128.13	** < 0.001**
CD4+CD28+/CD4+ (%)	87.12 ± 10.97	92.42 ± 8.95	** < 0.001**
CD4+CD28+ T cells (cells/ul)	600.64 ± 239.99	674.78 ± 271.48	**0.002**
CD8+CD28+/CD8+ (%)	50.95 ± 15.54	55.84 ± 17.26	**0.004**
CD8+CD28+ T cells (cells/ul)	249.24 ± 119.32	230.43 ± 106.65	0.156
CD8+HLA-DR+/CD8+ (%)	28.4 ± 10.7	38.37 ± 14.13	** < 0.001**
CD8+HLA-DR+ T cells (cells/ul)	148.75 ± 102.86	175.18 ± 129.29	**0.022**
CD8+CD38+/CD8+ (%)	22.34 ± 14.71	31.04 ± 13.35	** < 0.001**
CD8+CD38+ T cells (cells/ul)	114.11 ± 96.19	136.43 ± 95.69	**0.001**
CD4+/CD8+ (%)	1.62 ± 0.91	1.97 ± 1.00	** < 0.001**

Bold represents *p* <0.05, the difference was statistically significant

### Evaluation of relationships between lymphocyte subsets/myeloid cells and lung cancer stage

**Fig. 1:**
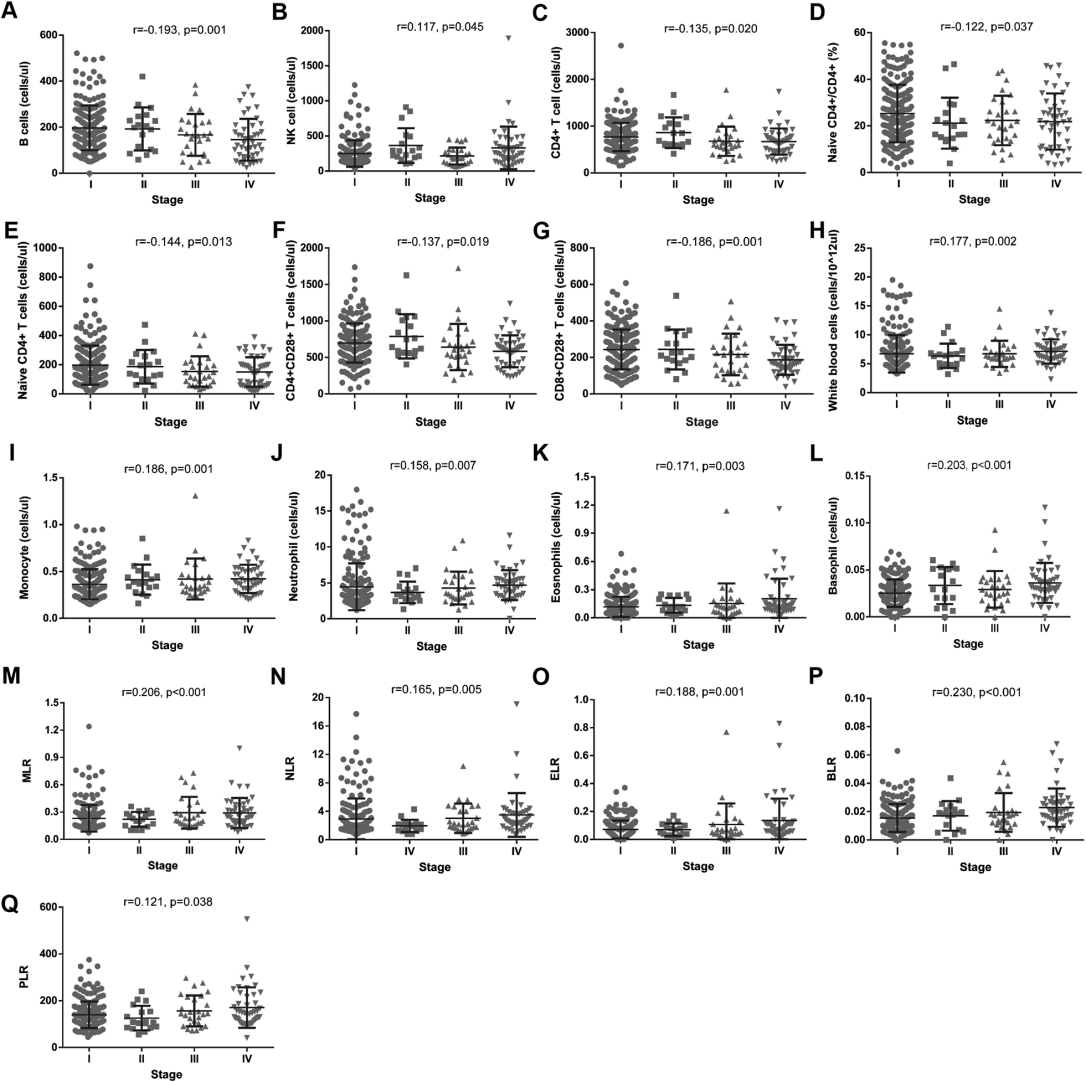
Predictive values of immune cell levels in progress of lung cancer. Distribution of B cell counts (**a**), NK cell counts (**b**), CD4+ T cell counts (**c**), naïve CD4+/CD4+ percentage, (**d**) naïve CD4+ T counts (**e**), CD4+CD28+ T counts (**f**), CD8+CD28+ T counts (**g**), WBC counts (**h**), monocytes counts (**i**), neutrophils counts (**j**), eosinophils counts (**k**), basophils counts (**l**), MLR (**m**), NLR (**n**), ELR (**o**), BLR (**p**), PLR (**q**)

To further analyze the role of immune cells in NSCLC progression, the 305 NSCLC patients were divided into 4 group by the stages. In this study, a trend of decrease in B cell counts (r = −0.193, *p* = 0.001, Fig. 1a), CD4+ T cell counts (r = −0.135, *p* = 0.020, Fig. 1c), naïve CD4+/CD4+ percentage (r = −0.122, *p* = 0.037, Fig. 1d), naïve CD4+ T cell counts (r = −0.144, *p* = 0.013, Fig. 1e), CD4+ CD28+ T cell counts (r = −0.137, *p* = 0.019, Fig. 1f), and CD8+CD28+ T cell counts (r = −0.186, *p* = 0.001, Fig. 1g) was noted for patients in advanced stages. In contrast,there were increasingly advanced stage related trend for NK cell counts (r = 0.117, *p* = 0.045, Fig. 1b), WBC counts (r = 0.177, *p* = 0.002, Fig. 1h), monocytes (r = 0.186, *p* = 0.001, Fig. 1i), neutrophils (r = 0.158 *p* = 0.007, Fig. 1j), eosinophils (r = 0.171, *p* = 0.003, Fig. 1k), basophils (r = 0.203, *p* < 0.001, Fig. 1l), MLR (r = 0.206, *p* < 0.001, Fig. 1m), NLR (r = 0.165, *p* = 0.005, Fig. 1n), ELR (r = 0.188, *p* = 0.001, Fig. 1o), BLR (r = 0.230, *p* < 0.001, Fig. 1p), PLR (r = 0.121, *p* = 0.038, Fig. 1q).There were no significant correlation between other immune cell levels and advanced stages (Additional file 1: Table S1). Notably, stage II patients had highest levels of T lymphocytes, NK cells, CD4+ T cells, CD8+ T cells, memory CD4+ T cells, CD4+CD28+ T cells, CD8+CD28+ T cells, CD8+HLA-DR+ T cells, lymphocytes and lowest counts of WBC, neutrophils than those patients in other stages.

### Assessment of relationships between lymphocyte subsets/myeloid cells and clinical parameters immune cell levels

**Fig. 2:**
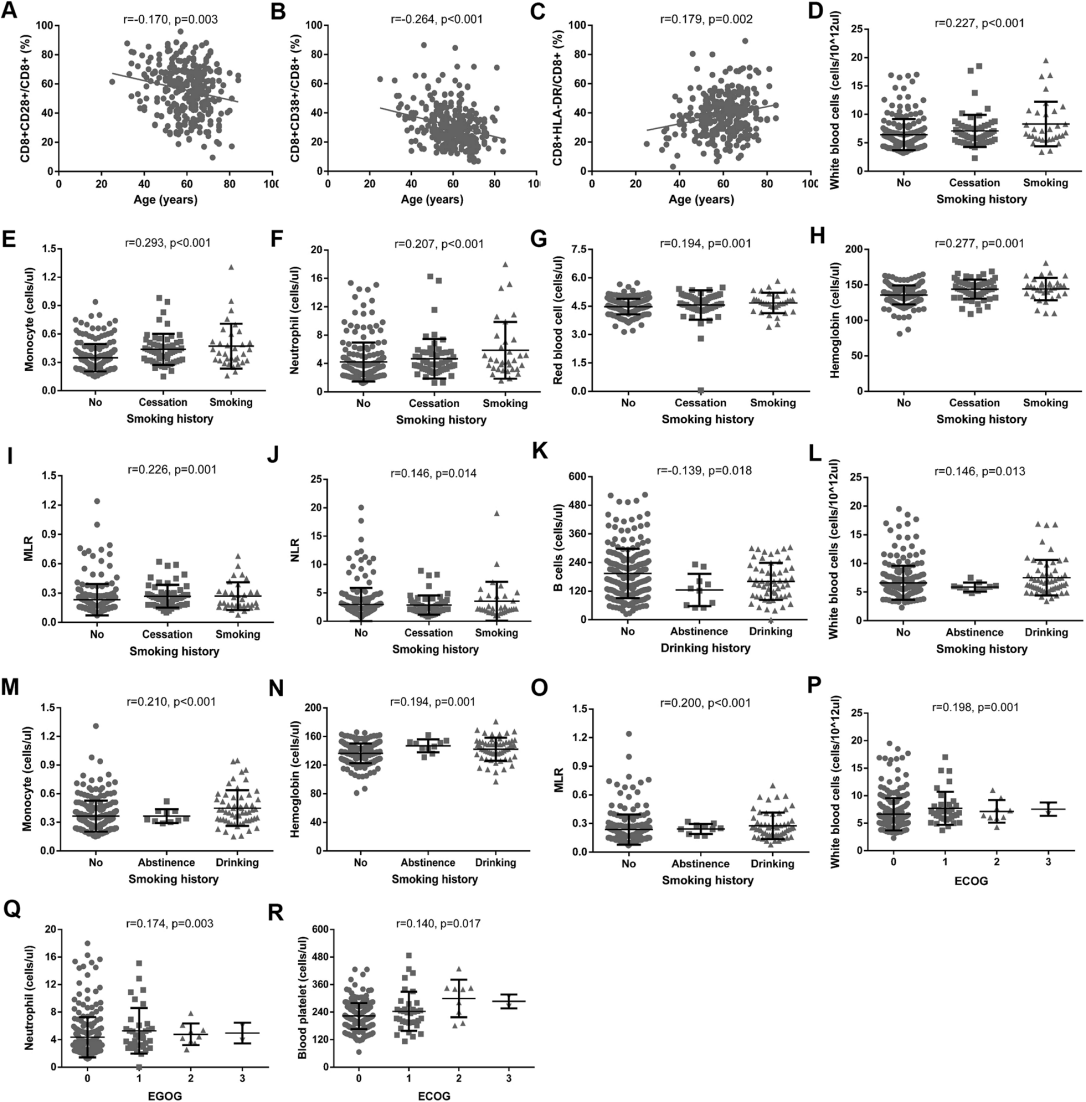
Relationship between immune cell levels and basic parameters for NSCLC patients. Age related change of CD8CD28/CD8+ percentage (**a**), CD8+CD38+/CD8+ percentage (**b**), CD8+HLA-DR+/CD8+ percentage (**c**); Smoking history related change of WBC counts (**d**), monocytes counts (**e**), neutrophils counts (**f**), RBC counts (**g**), hemoglobins counts (**h**), MLR (**i**), NLR (**j**); Drinking history related change of B cell counts (**k**), WBCcounts (**l**), monocytes counts (**m**), hemoglobins counts (**n**), MLR (**o**); ECOG related change of WBC counts (**p**), neutrophils counts (**q**), platelets counts (**r**)

To further demonstrate the relationship between immune cell levels and clinicopathologic characteristics we performed t text, Mann–Whitney test for 2 group, and Spearman’s rank correlation test for more than 2 groups, and the results weresummarized in Tables 3 and 4 and Fig. 2. There were high B cell counts (*p* < 0.001) and CD8+CD28+/CD8+ percentage (*p* = 0.047) in female patients than those in male. On the contrary, we discovered low counts of WBC (*p* = 0.005), monocytes (*p* < 0.001), neutrophils (*p* = 0.001), eosinophils (*p* = 0.006), RBC (*p* < 0.001), hemoglobins (*p* < 0.001), and MLR (*p* < 0.001), NLR (*p* = 0.001), ELR (*p* = 0.002), HLR (*p* = 0.007) in the female patients compared to those in the male patients. cell countsLow CD8+CD28+/CD8+ percentage (p = 0.008), CD4+/CD8+ ratio (p = 0.039), and high percentage of CD8+HLA-DR+ T cells (p = 0.019), CD8+CD38+/CD8+ (p = 0.016), CD8+CD38+ T cells (p = 0.013), RBC (p = 0.001), and hemoglobins (p < 0.001) were discovered in patients with surgery than patients without surgery. There were significant differences for memory CD4+/CD4+ percentage (*p* = 0.034), naïve CD4+/CD4+ percentage (*p* = 0.034), CD8+CD28+ T cells (*p* = 0.031), and monocytes (*p* = 0.002) in various histologies.

**Table 3:** Relationship between lymphocytes levels and clinicopathologic characteristics

	T lymphocytes (cells/ul)	B cells (cells/ul)	NK cells (cells/ul)	CD4+ T cells (cells/ul)	CD8+ T cells (cells/ul)	Memory CD4+/CD4+ (%)	Memory CD4+ T cells (cells/ul)	Naïve CD4+/ CD4+ (%)	Naïve CD4+ T cells (cells/ul)	CD4+CD28+/CD4+ (%)	CD4+CD28+ T cells (cells/ul)	CD8+CD28+/CD8+ (%)	CD8+CD28+ T cells (cells/ul)	CD8+HLA-DR/ CD8+ (%)	CD8+HLA-DR T cells (cells/ul)	CD8+CD38+/CD8+ (%)	CD8+CD38+ T cells (cells/ul)	CD4+/CD8+
Gender																		
Male	1220.79 ± 428.8	162.41 ± 92.49	294.41 ± 257.20	733.74 ± 329.37	443.59 ± 215.88	74.40 ± 13.31	532.37 ± 227.24	22.94 ± 11.97	169.68 ± 122.10	91.99 ± 9.66	649.61 ± 268.00	53.9 ± 17.42	224.69 ± 105.81	39.77 ± 14.11	184.25 ± 135.22	30.16 ± 13.31	135.34 ± 102.45	1.95 ± 1.14
Female	1277.29 ± 474.83	204.14 ± 99.30	247.43 ± 165.50	754.67 ± 278.59	435.51 ± 210.90	72.81 ± 12.50	544.86 ± 214.89	25.30 ± 12.32	195.75 ± 132.28	92.80 ± 8.30	696.41 ± 273.39	57.51 ± 16.99	235.36 ± 107.43	37.16 ± 14.07	167.39 ± 123.85	31.79 ± 13.37	137.36 ± 89.77	1.99 ± 0.88
P	0.431	** < 0.001**	0.318	0.287	0.657	0.226	0.226	0.560	0.122	0.067	0.360	0.120	**0.047**	0.401	0.084	0.129	0.203	0.141
Age																		
Yong	1220.60 ± 432.39	184.20 ± 96.01	239.06 ± 178.45	665.40 ± 311.44	464.27 ± 211.49	70.42 ± 12.80	450.16 ± 161.82	28.35 ± 12.75	207.01 ± 186.37	95.36 ± 4.36	611.66 ± 327.88	65.3 ± 13.68	288.07 ± 118.46	29.66 ± 11.17	143.33 ± 95.13	38.78 ± 13.86	174.12 ± 92.71	1.71 ± 0.92
Middle	1274.92 ± 452.69	193.84 ± 103.67	261.69 ± 203.82	745.08 ± 269.35	449.80 ± 219.66	73.16 ± 12.48	542.60 ± 217.98	25.27 ± 12.33	190.65 ± 122.15	92.76 ± 8.42	689.79 ± 256.35	57.03 ± 17.84	238.78 ± 102.11	37.58 ± 13.74	178.92 ± 133.65	32.53 ± 13.13	143.6 ± 86.67	1.90 ± 0.85
Elder	1239.03 ± 460.52	178.59 ± 94.77	279.02 ± 225.75	757.07 ± 323.13	427.96 ± 208.82	74.29 ± 13.17	550.16 ± 227.77	22.84 ± 11.88	175.23 ± 121.60	91.73 ± 9.73	673.78 ± 272.92	53.56 ± 16.83	215.74 ± 104.94	40.25 ± 14.32	177.39 ± 130.65	28.80 ± 12.90	125.61 ± 100.69	2.06 ± 1.11
P	0.688	0.550	0.446	0.152	0.425	0.275	0.077	0.055	0.338	0.121	0.169	**0.006**	**0.003**	**0.002**	0.521	** < 0.001**	**0.001**	0.123
Allergic history																		
Antibiotic	1247.95 ± 325.87	179.33 ± 111.83	213.09 ± 121.54	714.35 ± 243.96	447.59 ± 190.59	75.65 ± 11.83	533.42 ± 183.06	21.00 ± 11.73	159.85 ± 119.09	91.83 ± 10.97	640.66 ± 273.15	60.33 ± 17.27	250.68 ± 90.57	35.95 ± 15.01	169.99 ± 135.4	33.17 ± 16.32	146.63 ± 107.45	1.89 ± 1.07
Other	1397.30 ± 255.23	286.59 ± 60.68	245.97 ± 195.28	834.23 ± 210.96	475.6 ± 76.96	69.81 ± 9.10	590.88 ± 217.92	28.21 ± 10.03	226.12 ± 79.85	94.97 ± 2.95	795.26 ± 213.46	66.68 ± 9.31	320.55 ± 85.93	26.80 ± 5.40	126.96 ± 30.38	28.72 ± 11.96	137.68 ± 68.03	1.76 ± 0.36
No	1245.96 ± 478.24	184.37 ± 96.29	260.92 ± 195.87	752.53 ± 319.46	433.62 ± 209.25	73.03 ± 13.24	540.24 ± 231.06	24.81 ± 12.38	188.6 ± 131.45	92.42 ± 8.83	683.06 ± 276.73	55.43 ± 17.11	227.6 ± 109.03	38.18 ± 13.92	171.09 ± 120.46	30.92 ± 13.24	133.85 ± 91.74	1.99 ± 1.01
P	0.431	**0.018**	0.568	0.527	0.417	0.323	0.780	0.138	0.158	0.975	0.367	0.107	**0.026**	**0.033**	0.747	0.807	0.751	0.723
Surgery																		
Yes	1285.26 ± 471.23	182.04 ± 95.87	269.73 ± 203.91	749.71 ± 321.18	461.57 ± 221.21	73.04 ± 14.00	531.97 ± 210.35	24.12 ± 12.70	185.17 ± 136.17	91.56 ± 9.34	671.28 ± 269.38	53.28 ± 17.43	230.14 ± 108.07	39.62 ± 14.65	190.36 ± 136.55	32.75 ± 13.90	151.70 ± 106.33	1.88 ± 0.98
No	1231.11 ± 436.88	187.04 ± 98.35	245.17 ± 178.12	752.79 ± 294.67	421.08 ± 203.97	73.73 ± 11.76	553.58 ± 238.93	24.55 ± 11.76	186.15 ± 121.17	93.30 ± 8.54	687.78 ± 284.41	58.65 ± 17.11	234.36 ± 109.49	36.27 ± 13.26	158.90 ± 122.38	28.62 ± 11.73	118.04 ± 72.55	2.09 ± 1.07
P	0.428	0.866	0.272	0.708	0.098	0.898	0.456	0.552	0.653	0.141	0.543	**0.008**	0.593	0.072	**0.019**	**0.014**	**0.013**	**0.039**
History of diseases																		
No	1301.63 ± 413.78	187.44 ± 97.57	294.99 ± 271.76	752.48 ± 256.8	462.89 ± 233.94	73.22 ± 12.76	545.68 ± 193.04	25.08 ± 12.72	193.87 ± 139.93	91.08 ± 8.93	686.59 ± 249.44	55.50 ± 16.87	238.56 ± 106.90	38.49 ± 14.95	185.52 ± 142.82	33.55 ± 12.46	154.74 ± 96.26	2.00 ± 1.07
Yes	1234.46 ± 470.19	183.60 ± 96.63	252.92 ± 188.98	747.32 ± 322.23	431.22 ± 204.75	73.54 ± 13.19	539.69 ± 233.47	23.99 ± 12.24	181.21 ± 124.61	93.01 ± 8.92	674.79 ± 283.1	56.31 ± 17.74	229.26 ± 108.56	38.13 ± 14.15	170.82 ± 125.08	30.13 ± 13.64	128.74 ± 90.76	1.98 ± 1.00
P	0.318	0.670	0.196	0.600	0.453	0.808	0.566	0.601	0.468	**0.043**	0.629	0.644	0.541	0.741	0.455	**0.012**	**0.022**	0.864
Smoking history																		
No	1249.36 ± 463.71	194.16 ± 98.74	240.88 ± 167.3	744.68 ± 317.46	436.33 ± 214.38	72.58 ± 13.3	528.07 ± 217.8	25.19 ± 12.45	191.36 ± 137.93	92.66 ± 8.77	672.86 ± 272.05	55.95 ± 17.32	228.75 ± 105.67	37.11 ± 13.89	168.54 ± 130.16	31.02 ± 12.18	134.95 ± 87.04	1.95 ± 0.97
Yes	1395.45 ± 460.14	204.81 ± 106.52	267.17 ± 174.27	854.83 ± 322.1	471.48 ± 185.99	74.52 ± 11.83	541.63 ± 201.09	22.63 ± 11.47	167.39 ± 103.98	91.2 ± 9.42	653.13 ± 253.27	54.51 ± 15.95	228.12 ± 105.94	39.96 ± 14.98	185.87 ± 139.02	30.78 ± 14.5	138.87 ± 111.4	2.01 ± 0.86
Cessation	1220.78 ± 388.34	151.82 ± 90.12	329.4 ± 260.19	728.75 ± 246.2	441.08 ± 209.86	75.07 ± 12.07	634.99 ± 265.64	23.24 ± 12.14	204.1 ± 122.55	93.16 ± 9.43	791.84 ± 313.28	58.35 ± 18.73	264.2 ± 115.09	37.68 ± 13.03	185.99 ± 109.14	26.63 ± 10.94	122.51 ± 78.83	2.05 ± 1.17
P	0.124	**0.007**	0.170	0.086	0.343	0.527	0.062	0.382	0.377	0.136	0.088	0.638	0.200	0.437	0.179	0.133	0.767	0.882
Drinking history																		
No	1261.05 ± 464.45	194.59 ± 103.37	248.44 ± 175.23	752.68 ± 323.32	451.27 ± 223.03	73.12 ± 13.24	541.44 ± 234.83	24.47 ± 12.37	185.61 ± 130.73	92.61 ± 9.08	678.53 ± 287.05	55.91 ± 17.39	237.64 ± 113.07	38.02 ± 14.7	180.04 ± 139.59	31.42 ± 13.64	140.47 ± 95.31	1.92 ± 0.98
Yes	1261.86 ± 459.97	160.90 ± 77.59	303.90 ± 251.43	762.52 ± 255.07	409.57 ± 187.12	77.26 ± 14.85	528.60 ± 176.76	20.22 ± 14.72	151.40 ± 132.36	91.04 ± 6.82	636.21 ± 206.03	55.11 ± 12.18	231.52 ± 67.87	37.27 ± 9.00	154.28 ± 35.43	29.04 ± 10.35	120.53 ± 43.69	2.22 ± 1.13
Abstinence	1193.52 ± 242.43	124.90 ± 67.65	219.10 ± 116.42	698.20 ± 219.11	426.90 ± 104.67	72.98 ± 12.51	547.67 ± 185.77	25.24 ± 12.38	200.74 ± 131.11	91.99 ± 8.7	699.64 ± 244.28	55.65 ± 17.8	212.66 ± 90.33	38.86 ± 13.23	162.34 ± 104.68	29.20 ± 12.78	122.15 ± 94.93	1.76 ± 0.80
P	0.902	**0.020**	0.651	0.589	0.542	0.454	0.738	0.364	0.312	0.291	0.626	0.937	0.438	0.676	0.925	0.458	0.177	0.152
ECOG PS																		
0	1249.01 ± 470.92	189.54 ± 97.22	256.76 ± 188.20	755.79 ± 314.37	433.64 ± 208.84	73.07 ± 13.01	542.99 ± 227.84	24.55 ± 12.23	188.23 ± 130.96	92.41 ± 9.11	687.18 ± 275.96	56.88 ± 17.01	233.15 ± 108.53	37.34 ± 14.28	168.51 ± 125.77	30.31 ± 12.37	129.85 ± 84.49	2.00 ± 1.00
1	1269.76 ± 376.02	157.46 ± 88.47	314.7 ± 345.09	723.17 ± 241.1	463.12 ± 259.22	74.22 ± 12.16	531.84 ± 187.04	24.57 ± 11.79	181.96 ± 110.51	93.97 ± 5.72	638.85 ± 261.14	51.87 ± 18.1	214.82 ± 99.14	40.64 ± 13.28	194.72 ± 158.1	33.24 ± 15.22	161.51 ± 137.98	2.02 ± 1.15
2	1202.74 ± 242.49	181.56 ± 165.45	390.44 ± 282.96	632.00 ± 218.54	422.78 ± 129.95	77.45 ± 16.10	462.00 ± 79.90	19.88 ± 15.63	153.11 ± 176.08	88.14 ± 14.21	572.20 ± 257.73	49.58 ± 18.76	199.06 ± 87.64	44.74 ± 13.11	197.60 ± 117.35	38.93 ± 18.6	177.33 ± 133.09	1.58 ± 0.57
3	1268 ± 534.57	257 ± 96.17	312 ± 91.92	633.5 ± 297.69	589 ± 260.22	90.59 ± 3.75	579.5 ± 293.45	7.69 ± 4.51	42.00 ± 5.66	96.05 ± 0.49	608 ± 282.84	52.1 ± 10.04	294 ± 76.37	45.05 ± 13.51	283 ± 196.58	20.95 ± 7.42	133.5 ± 98.29	1.07 ± 0.04
P	0.966	0.093	0.267	0.541	0.776	0.138	0.779	0.115	0.075	0.881	0.617	0.264	0.395	0.104	0.427	0.280	0.705	0.188
Histology																		
LAC	1239.83 ± 463.04	186.72 ± 98.1	262.97 ± 207.77	741.78 ± 309.04	432.47 ± 215.11	73.16 ± 12.97	533.68 ± 223.57	24.59 ± 12.26	185.23 ± 129.68	92.36 ± 9.22	669.8 ± 275.26	55.87 ± 17.38	226.45 ± 107.45	38.19 ± 14.1	171.93 ± 129.37	31.34 ± 12.95	135.01 ± 90.69	2.00 ± 1.03
LSC	1355.94 ± 346.96	161.44 ± 98.52	308.93 ± 241.53	766.33 ± 231.97	508.07 ± 182.7	78.18 ± 10.83	593.22 ± 184.44	19.62 ± 10.23	157.22 ± 97.15	92.73 ± 5.56	711.58 ± 223.72	54.83 ± 16.04	266.91 ± 91.29	40.11 ± 14.84	208.43 ± 128.57	28.67 ± 16.78	153.82 ± 138.5	1.69 ± 0.73
LASC	1562.36	298.00	909.00	1060.00	459.00	54.34	576.00	44.72	474.00	100.00	1060	75.90	348.38	38.70	177.63	12.60	57.83	2.31
P	0.189	0.111	0.206	0.299	0.071	**0.034**	0.189	**0.034**	0.178	0.132	0.185	0.430	**0.031**	0.908	0.121	0.072	0.492	0.336
Tumor stage																		
T1	1287.03 ± 461.46	199.48 ± 97.84	251.40 ± 185.00	771.03 ± 306.11	452.45 ± 209.34	72.5 ± 12.94	548.64 ± 209.38	25.08 ± 12.12	195.9 ± 134.25	92.84 ± 8.03	700.98 ± 270.52	55.8 ± 16.78	241.11 ± 109.81	37.68 ± 13.55	175.94 ± 127.54	29.33 ± 11.65	132.03 ± 81.94	1.93 ± 0.92
T2	1132.15 ± 467.27	151.1 ± 86.35	237.71 ± 174.92	651.4 ± 307.11	393.92 ± 221.18	75.83 ± 11.66	486.99 ± 232.91	22.24 ± 11.46	151.16 ± 110.95	91.5 ± 9.2	580.82 ± 302.24	57.08 ± 17.45	206.53 ± 106.04	38.86 ± 14.46	161.27 ± 128.67	32.53 ± 14.45	128.38 ± 103.22	2.00 ± 1.03
T3	1249.3 ± 474.78	138.25 ± 67.27	306.25 ± 230.88	766.37 ± 303.9	410.42 ± 206.36	78.33 ± 10.56	598.67 ± 258.33	20.01 ± 10.82	155.53 ± 92.08	93.41 ± 5.16	715.91 ± 297.6	59.04 ± 16.63	226.78 ± 116.11	36.41 ± 13.99	164.79 ± 133.54	29.23 ± 13.01	120.75 ± 104.33	2.17 ± 0.80
T4	1226.94 ± 342.63	167.96 ± 107.62	349.64 ± 223.54	725.14 ± 207.8	427 ± 229.09	75.31 ± 12.43	541.55 ± 176.54	22.98 ± 12.26	170.36 ± 102.28	92.89 ± 9.07	670.33 ± 195.47	53.65 ± 21.62	199.97 ± 80.2	40.49 ± 14.87	173.75 ± 117.93	35.83 ± 15.59	163.63 ± 144.56	2.17 ± 1.28
P	0.085	**0.002**	0.093	**0.033**	0.257	0.142	0.100	0.188	0.129	0.923	**0.016**	0.702	0.088	0.612	0.669	0.191	0.430	0.532
Lymph nodes metastases																		
N0	1273.8 ± 446.29	195.1 ± 97.38	251.71 ± 181.65	768.04 ± 303.92	441.54 ± 198.47	72.86 ± 12.89	549.78 ± 211.12	24.95 ± 12.09	193.59 ± 131.95	93.11 ± 7.56	697.13 ± 272.62	57.11 ± 16.46	241.44 ± 109.47	37.11 ± 13.59	168.68 ± 117.45	29.45 ± 11.66	128.97 ± 77.58	1.96 ± 0.93
N1	1400.43 ± 633.16	212.75 ± 108.69	282.25 ± 206.57	779.17 ± 316.94	472.25 ± 267.66	76.96 ± 13.38	583.42 ± 222.68	21.28 ± 13.32	179.58 ± 140.93	90.83 ± 11.91	698.56 ± 266.3	51.27 ± 20.07	215.31 ± 91.73	40.2 ± 12.8	197.74 ± 138.02	33.98 ± 19.63	155.51 ± 121.06	1.91 ± 0.86
N2	1241.51 ± 463.87	158.63 ± 85.08	254.33 ± 146.18	686.63 ± 320.14	477.71 ± 276.70	75.19 ± 11.33	511.32 ± 248.97	21.63 ± 10.81	154.41 ± 107.8	90.83 ± 9.74	630.08 ± 318.86	50.32 ± 17.87	217.47 ± 116.06	41.15 ± 14.88	213.69 ± 183.42	30.12 ± 14.02	149.22 ± 131.7	1.84 ± 1.01
N3	1068.74 ± 429.88	136.71 ± 91.75	271.63 ± 179.01	650.58 ± 231.06	360.04 ± 211.56	74.49 ± 13.07	478.46 ± 182.73	23.75 ± 12.99	161.13 ± 110.39	91.98 ± 9.08	595.73 ± 212.16	56.8 ± 20.42	180.36 ± 78.89	40.46 ± 14.02	147.53 ± 108.87	36.11 ± 13.48	140.09 ± 133.64	2.23 ± 1.22
P	0.248	**0.008**	0.729	0.127	0.115	0.545	0.227	0.385	0.266	0.556	0.152	0.202	**0.030**	0.217	0.492	0.114	0.936	0.696
Distant metastases																		
M0	1281.78 ± 462.6	193.06 ± 97.13	254.16 ± 190.11	763.80 ± 308.98	448.96 ± 214.58	73.1 ± 12.83	548.94 ± 218.46	24.6 ± 12.05	190.51 ± 131	93.00 ± 7.89	694.48 ± 279.02	56.38 ± 16.78	239.97 ± 110.12	37.69 ± 13.94	175.8 ± 131.19	29.61 ± 12.1	132.35 ± 87.17	1.94 ± 0.91
M1	1141.48 ± 396.68	145.72 ± 90.52	330.67 ± 302.36	671.32 ± 279.37	397.7 ± 198.3	75.95 ± 12.17	507.09 ± 242.88	21.89 ± 12.04	149.67 ± 101.18	88.73 ± 13.12	583.34 ± 219.81	52.2 ± 18.79	186.2 ± 81.8	40.76 ± 12.99	168.68 ± 109.18	35.05 ± 15.59	146.02 ± 121.53	2.12 ± 1.40
P	0.099	**0.001**	**0.049**	**0.040**	0.121	0.278	0.103	0.208	**0.049**	**0.040**	**0.015**	0.099	**0.001**	0.065	0.827	0.053	0.908	0.926

Bold represents *p* <0.05, the difference was statistically significant

*LAC* lung adenocarcinoma, *LSC* squamous carcinoma, *LASC* lung adenosquamous carcinoma

**Table 4:** Relationship between inflammatory cells levels and clinicopathologic characteristics

	WBC (cells/10^12ul)	Lymphocytes (cells/10^12ul)	Monocytes (cells/ul)	Neutrophils (cells/ul)	Eosnophils (cells/ul)	Basophils (cells/ul)	RBC (cells/ul)	Hemoglobins (cells/ul)	Blood platelets (cells/ul)	MLR	NLR	ELR	BLR	RLR	HLR	PLR
Gender																
Male	7.10 ± 2.99	1.71 ± 0.57	0.44 ± 0.18	4.79 ± 2.98	0.15 ± 0.15	0.03 ± 0.02	4.68 ± 0.61	144.94 ± 13.64	223.76 ± 63.84	0.28 ± 0.15	3.18 ± 2.6	0.13 ± 0.43	0.03 ± 0.13	3.07 ± 1.38	96.81 ± 45.58	146.20 ± 78.14
Female	6.42 ± 2.79	1.76 ± 0.62	0.33 ± 0.14	4.14 ± 2.81	0.12 ± 0.13	0.03 ± 0.02	4.37 ± 0.51	132.6 ± 12.14	231.43 ± 62.2	0.21 ± 0.15	2.82 ± 2.86	0.07 ± 0.08	0.02 ± 0.01	2.82 ± 1.21	85.59 ± 36.44	146.73 ± 63.55
* P*	**0.005**	0.693	** < 0.001**	**0.001**	**0.006**	0.574	** < 0.001**	** < 0.001**	0.217	** < 0.001**	**0.001**	**0.002**	0.649	0.054	**0.007**	0.570
Age																
Yong	6.39 ± 2.91	1.79 ± 0.84	0.37 ± 0.14	3.99 ± 2.2	0.1 ± 0.09	0.02 ± 0.01	4.39 ± 0.59	130.24 ± 21.42	231.48 ± 73.2	0.23 ± 0.12	2.81 ± 3.3	0.07 ± 0.07	0.01 ± 0.01	2.89 ± 1.27	85.83 ± 41.37	150.75 ± 76.23
Middle	7.06 ± 3.14	1.75 ± 0.58	0.38 ± 0.17	4.77 ± 3.26	0.14 ± 0.18	0.03 ± 0.02	4.6 ± 0.45	140.82 ± 13.48	235.03 ± 58.01	0.24 ± 0.15	3.29 ± 3.21	0.12 ± 0.47	0.03 ± 0.15	2.96 ± 1.36	92.60 ± 46.07	153.95 ± 87.09
Elder	6.55 ± 2.71	1.72 ± 0.57	0.38 ± 0.16	4.27 ± 2.71	0.13 ± 0.11	0.03 ± 0.01	4.47 ± 0.65	137.71 ± 12.93	222.26 ± 64.53	0.25 ± 0.15	2.81 ± 2.26	0.09 ± 0.09	0.02 ± 0.01	2.93 ± 1.25	90.19 ± 37.56	140.53 ± 54.57
* P*	0.204	0.868	0.948	0.243	0.198	0.385	**0.029**	**0.010**	0.148	0.766	0.401	0.157	0.278	0.998	0.718	0.693
Allergic history																
Antibiotic	6.48 ± 3.01	1.68 ± 0.44	0.36 ± 0.15	4.29 ± 2.91	0.15 ± 0.22	0.03 ± 0.02	4.39 ± 0.93	137.84 ± 13.4	217.74 ± 50.39	0.22 ± 0.09	2.64 ± 1.77	0.09 ± 0.12	0.02 ± 0.01	2.81 ± 0.95	87.96 ± 25.82	138.27 ± 54.36
Other	8.45 ± 5.03	1.94 ± 0.19	0.44 ± 0.27	6.01 ± 5.12	0.13 ± 0.12	0.02 ± 0.01	4.51 ± 0.34	132.83 ± 18.74	196.17 ± 47.63	0.23 ± 0.13	3.06 ± 2.56	0.06 ± 0.06	0.01 ± 0.01	2.33 ± 0.19	68.34 ± 7.28	101.13 ± 24.55
No	6.75 ± 2.95	1.72 ± 0.63	0.38 ± 0.17	4.46 ± 2.97	0.13 ± 0.13	0.03 ± 0.02	4.54 ± 0.46	138.65 ± 14.4	228.96 ± 64.09	0.25 ± 0.16	3.11 ± 2.98	0.08 ± 0.1	0.02 ± 0.01	3.02 ± 1.38	93.26 ± 44.67	150.07 ± 74.34
* P*	0.413	0.315	0.750	0.553	0.782	0.730	0.891	0.665	0.373	0.777	0.957	0.893	0.409	0.363	0.214	0.096
Surgery history																
Yes	6.97 ± 3.2	1.75 ± 0.59	0.4 ± 0.18	4.65 ± 3.25	0.14 ± 0.15	0.03 ± 0.02	4.57 ± 0.6	140.82 ± 15.29	229.88 ± 62.68	0.25 ± 0.16	3.13 ± 3	0.09 ± 0.1	0.02 ± 0.01	2.99 ± 1.43	91.69 ± 43.48	147.11 ± 72.87
No	6.53 ± 2.72	1.72 ± 0.61	0.37 ± 0.15	4.26 ± 2.7	0.12 ± 0.14	0.03 ± 0.02	4.46 ± 0.43	135.53 ± 12.88	223.73 ± 62.25	0.24 ± 0.14	2.88 ± 2.61	0.08 ± 0.09	0.02 ± 0.01	2.94 ± 1.19	89.57 ± 36.65	142.06 ± 53.96
* P*	0.273	0.422	0.305	0.190	0.113	0.640	**0.016**	**0.001**	0.392	0.248	0.290	0.125	0.802	0.961	0.932	0.724
History of diseases																
Yes	6.78 ± 3.04	1.84 ± 0.66	0.38 ± 0.16	4.39 ± 2.96	0.13 ± 0.16	0.03 ± 0.02	4.59 ± 0.44	139.17 ± 13.08	224.72 ± 59.42	0.23 ± 0.13	2.74 ± 2.48	0.08 ± 0.1	0.02 ± 0.01	2.77 ± 0.95	84.33 ± 29.28	134.86 ± 55.33
No	6.73 ± 2.95	1.69 ± 0.59	0.38 ± 0.17	4.48 ± 2.99	0.13 ± 0.14	0.03 ± 0.02	4.5 ± 0.56	137.9 ± 14.86	227.4 ± 64.64	0.25 ± 0.16	3.13 ± 2.93	0.09 ± 0.1	0.02 ± 0.01	3.03 ± 1.41	93.82 ± 45.67	151.01 ± 76.92
* P*	0.624	0.158	0.909	0.276	0.558	0.640	0.484	0.732	0.734	0.128	0.058	0.315	0.124	0.366	0.354	0.164
Smoking history																
No	6.45 ± 2.74	1.72 ± 0.61	0.35 ± 0.14	4.22 ± 2.75	0.13 ± 0.14	0.03 ± 0.02	4.48 ± 0.41	135.79 ± 13.4	226.4 ± 61.11	0.23 ± 0.16	2.96 ± 2.92	0.08 ± 0.1	0.02 ± 0.01	2.98 ± 1.27	90.32 ± 39.30	146.68 ± 62.1
Yes	8.29 ± 3.92	1.89 ± 0.57	0.47 ± 0.24	5.87 ± 4.01	0.13 ± 0.09	0.03 ± 0.02	4.67 ± 0.54	144.21 ± 15.72	238.48 ± 69.31	0.27 ± 0.14	3.53 ± 3.42	0.22 ± 0.86	0.06 ± 0.27	2.86 ± 1.72	88.19 ± 52.90	143.81 ± 87.75
Cessation	7.1 ± 2.83	1.75 ± 0.53	0.44 ± 0.16	4.6 ± 2.87	0.15 ± 0.12	0.03 ± 0.02	4.56 ± 0.78	143.95 ± 13.42	227.61 ± 65.45	0.27 ± 0.16	2.85 ± 1.69	0.09 ± 0.07	0.02 ± 0.01	2.88 ± 1.10	91.01 ± 32.92	139.32 ± 50.79
* P*	**0.001**	0.121	** < 0.001**	**0.002**	0.098	0.145	**0.004**	** < 0.001**	0.810	** < 0.001**	**0.039**	0.145	0.493	0.291	0.436	0.544
Drinking history																
No	6.61 ± 2.96	1.74 ± 0.62	0.36 ± 0.16	4.35 ± 2.96	0.13 ± 0.13	0.03 ± 0.02	4.49 ± 0.52	136.56 ± 13.76	226.67 ± 65.26	0.24 ± 0.16	2.96 ± 2.83	0.08 ± 0.09	0.02 ± 0.01	2.95 ± 1.30	90.67 ± 42.41	146.45 ± 71.94
Yes	7.5 ± 3.11	1.76 ± 0.58	0.45 ± 0.19	5.06 ± 3.21	0.13 ± 0.14	0.03 ± 0.02	4.58 ± 0.54	142.33 ± 16.02	237.23 ± 56.72	0.28 ± 0.14	3.37 ± 2.99	0.17 ± 0.66	0.04 ± 0.21	2.93 ± 1.43	90.94 ± 43.80	150.91 ± 75.76
Abstinence	5.88 ± 0.78	1.55 ± 0.37	0.36 ± 0.07	3.71 ± 0.67	0.15 ± 0.08	0.03 ± 0.01	4.64 ± 0.3	147 ± 8.94	202.7 ± 44.87	0.24 ± 0.05	2.52 ± 0.83	0.11 ± 0.07	0.02 ± 0.01	3.14 ± 0.75	99.63 ± 23.28	137.85 ± 43.66
* P*	**0.026**	0.608	**0.002**	0.066	0.399	0.747	0.267	**0.002**	0.224	**0.003**	0.062	0.227	0.511	0.391	0.208	0.860
ECOG																
0	6.63 ± 2.95	1.73 ± 0.61	0.37 ± 0.16	4.35 ± 2.93	0.13 ± 0.13	0.03 ± 0.02	4.52 ± 0.6	138.95 ± 13.76	223.09 ± 56.85	0.24 ± 0.15	2.95 ± 2.77	0.1 ± 0.33	0.02 ± 0.1	2.97 ± 1.35	9.20 ± 43.06	144.96 ± 71.59
1	7.69 ± 3.02	1.76 ± 0.58	0.44 ± 0.21	5.28 ± 3.31	0.15 ± 0.21	0.03 ± 0.02	4.52 ± 0.54	135.51 ± 17.79	243.46 ± 85.24	0.28 ± 0.16	3.67 ± 3.27	0.09 ± 0.12	0.02 ± 0.01	2.85 ± 1.09	85.63 ± 34.63	153.77 ± 74.35
2	7.14 ± 2.05	1.78 ± 0.56	0.41 ± 0.09	4.78 ± 1.57	0.12 ± 0.03	0.03 ± 0.01	4.41 ± 0.47	128.44 ± 15.29	299.33 ± 81.73	0.25 ± 0.06	2.79 ± 0.89	0.07 ± 0.03	0.02 ± 0	2.7 ± 0.91	78.28 ± 25.33	177.7 ± 58.41
3	7.52 ± 1.23	1.85 ± 0.54	0.39 ± 0.09	4.96 ± 1.5	0.27 ± 0.17	0.05 ± 0.02	4.79 ± 0.2	145.5 ± 6.36	286.5 ± 30.41	0.23 ± 0.11	2.92 ± 1.66	0.17 ± 0.14	0.03 ± 0.02	2.72 ± 0.90	82.63 ± 27.44	159.19 ± 29.81
* P*	**0.008**	0.974	0.069	**0.027**	0.426	0.146	0.448	0.116	**0.013**	0.207	0.146	0.670	0.357	0.949	0.714	0.206
Histology																
LAC	6.65 ± 2.88	1.72 ± 0.61	0.37 ± 0.15	4.39 ± 2.91	0.13 ± 0.14	0.03 ± 0.02	4.52 ± 0.59	138.32 ± 14.25	227.25 ± 63.48	0.24 ± 0.15	3.02 ± 2.84	0.08 ± 0.1	0.02 ± 0.01	2.98 ± 1.33	92.06 ± 42.41	147.99 ± 72.41
LSC	7.5 ± 2.99	1.85 ± 0.48	0.5 ± 0.24	4.9 ± 2.89	0.17 ± 0.12	0.03 ± 0.01	4.45 ± 0.42	136.85 ± 13.66	234.58 ± 59.29	0.28 ± 0.14	2.68 ± 1.43	0.27 ± 0.95	0.07 ± 0.3	2.44 ± 0.84	78.06 ± 22.37	132.90 ± 44.88
LASC	10.30	2.78	0.85	6.29	0.12	0.05	5.17	163.00	234.00	0.31	2.26	0.04	0.02	1.86	58.63	84.17
* P*	0.076	0.089	**0.002**	0.153	0.078	0.223	0.173	0.233	0.864	0.073	0.787	0.148	0.918	0.090	0.106	0.281
Tumor stage																
T1	6.73 ± 3.1	1.78 ± 0.6	0.37 ± 0.16	4.46 ± 3.18	0.12 ± 0.11	0.03 ± 0.02	4.54 ± 0.43	139.19 ± 12.52	220.86 ± 50.95	0.23 ± 0.15	2.93 ± 2.89	0.07 ± 0.06	0.02 ± 0.01	2.87 ± 1.16	87.98 ± 35.62	136.47 ± 54.37
T2	6.07 ± 2.75	1.5 ± 0.61	0.37 ± 0.17	3.89 ± 2.51	0.15 ± 0.19	0.03 ± 0.02	4.39 ± 0.83	136.91 ± 16.88	212.38 ± 72.67	0.28 ± 0.17	2.9 ± 2.14	0.12 ± 0.16	0.02 ± 0.01	3.45 ± 1.65	106.71 ± 49.24	158.63 ± 68.37
T3	7.6 ± 2.69	1.71 ± 0.6	0.5 ± 0.25	5.22 ± 2.49	0.13 ± 0.08	0.04 ± 0.02	4.58 ± 0.59	137.56 ± 17.66	275.56 ± 97.81	0.32 ± 0.17	3.86 ± 4.25	0.38 ± 1.23	0.12 ± 0.39	3.18 ± 2.23	96.30 ± 69.57	184.22 ± 116.91
T4	7.48 ± 1.63	1.76 ± 0.4	0.44 ± 0.14	4.77 ± 1.77	0.25 ± 0.25	0.04 ± 0.02	4.61 ± 0.46	136.77 ± 16.46	264.46 ± 62.89	0.27 ± 0.1	3.03 ± 1.15	0.15 ± 0.15	0.03 ± 0.02	2.77 ± 0.72	81.86 ± 21.57	160.05 ± 60.92
* P*	**0.001**	**0.012**	**0.001**	**0.005**	**0.010**	** < 0.001**	0.793	0.763	** < 0.001**	**0.001**	**0.009**	**0.004**	**0.001**	0.079	0.055	0.054
Lymph nodes metastasesLymph nodes metastases																
N0	6.66 ± 3.19	1.76 ± 0.6	0.37 ± 0.16	4.42 ± 3.2	0.12 ± 0.11	0.03 ± 0.02	4.52 ± 0.43	138.51 ± 12.6	221.37 ± 54.06	0.23 ± 0.14	2.88 ± 2.8	0.07 ± 0.06	0.02 ± 0.01	2.89 ± 1.17	88.64 ± 36.07	137.98 ± 54.92
N1	6.37 ± 1.86	1.91 ± 0.78	0.38 ± 0.17	3.8 ± 1.4	0.19 ± 0.31	0.04 ± 0.03	4.55 ± 0.47	136.08 ± 20.46	242.17 ± 53.11	0.21 ± 0.08	2.29 ± 1.24	0.11 ± 0.18	0.02 ± 0.02	2.72 ± 1.01	80.3 ± 30.07	146.62 ± 65.04
N2	6.92 ± 2.37	1.61 ± 0.56	0.42 ± 0.21	4.56 ± 2.33	0.16 ± 0.2	0.03 ± 0.02	4.58 ± 0.47	137.76 ± 15.24	233.91 ± 87.64	0.29 ± 0.18	3.23 ± 2.12	0.1 ± 0.14	0.02 ± 0.01	3.23 ± 1.36	97.25 ± 42.04	158.95 ± 71.24
N3	7.18 ± 1.85	1.52 ± 0.46	0.45 ± 0.14	4.67 ± 2.21	0.19 ± 0.17	0.04 ± 0.02	4.74 ± 0.46	143.75 ± 17.54	246.29 ± 69.55	0.34 ± 0.19	4.05 ± 3.96	0.35 ± 1	0.09 ± 0.32	3.61 ± 2.06	109.83 ± 64.92	181.93 ± 98.02
* P*	**0.020**	0.238	**0.010**	0.096	0.108	0.041	0.275	0.447	0.284	** < 0.001**	**0.007**	**0.012**	**0.001**	0.093	0.124	**0.048**
Distant metastases																
M0	6.68 ± 3.08	1.76 ± 0.61	0.37 ± 0.17	4.4 ± 3.1	0.12 ± 0.12	0.03 ± 0.02	4.52 ± 0.42	138.12 ± 13.03	221.55 ± 54.66	0.24 ± 0.15	2.89 ± 2.72	0.07 ± 0.08	0.02 ± 0.01	2.91 ± 1.19	88.82 ± 36.55	139.34 ± 56.81
M1	7.13 ± 2.1	1.65 ± 0.53	0.42 ± 0.15	4.7 ± 2.09	0.2 ± 0.21	0.04 ± 0.02	4.69 ± 0.56	140.78 ± 18.37	255.59 ± 88.44	0.29 ± 0.17	3.49 ± 3.07	0.24 ± 0.73	0.06 ± 0.23	3.26 ± 1.73	98.76 ± 54.84	170.25 ± 86.91
* P*	**0.003**	0.415	**0.011**	**0.004**	**0.003**	**0.001**	**0.034**	0.183	**0.013**	**0.003**	**0.002**	** < 0.001**	** < 0.001**	0.233	0.352	**0.011**

Bold represents *p* <0.05, the difference was statistically significant

*LAC* lung adenocarcinoma, *LSC* squamous carcinoma, *LASC* lung adenosquamous carcinoma

A trend of decreased CD8+CD28+/CD8+ percentage (r = −0.170, *p* = 0.006, Fig. 2a), CD8+CD38+/CD8+ percentage (r = −0.264, *p* < 0.001, Fig. 2b), and increased CD8+HLA-DR+/CD8+ percentage (r = 0.179, *p* = 0.002, Fig. 2c) with age was found in our study. However, we did not find asimilar trend in RBC and hemoglobins in spite of statistically significant difference (r = −0.047, *p* = 0.416; r = 0.004, *p* = 0.943) for these data. There were increased WBC (r = 0.227, *p* < 0.001, Fig. 2d), monocytes (r = 0.293, *p* < 0.001, Fig. 2e), neutrophils (r = 0.207, *p* < 0.001, Fig. 2f), RBC (r = 0.194, *p* = 0.001, Fig. 2g), hemoglobins (r = 0.277, *p* < 0.001, Fig. 2h), and MLR (r = 0.226, *p* < 0.001, Fig. 2i), NLR (r = 0.150, *p* = 0.011, Fig. 2j) with in patientswith various smoking history statuses. In addition, we also found patients with smoking cessation had lower B cell counts (r = −0.082, *p* = 0.166) compared to that in patients with smoking or without smoking. There wasa decreased trend in B cell counts (r = −0.139, *p* = 0.018, Fig. 2k) and increased trend in WBC (r = 0.146, *p* = 0.013, Fig. 2l), monocyte counts (r = 210, *p* < 0.001, Fig. 2m), hemoglobin counts (r = 0.194, *p* = 0.001, Fig. 2n) and MLR (r = 0.200, *p* < 0.001, Fig. 2o) with in patients with various drinking history statuses. A trend of an increase in WBC (r = 0.198, *p* = 0.001, Fig. 2p), neutrophils (r = 0.174, *p* = 0.003, Fig. 2q), and platelets (r = 0.140, *p* = 0.017, Fig. 2r) was found with increased ECOG. In the lung cancer cohorts, we discovered that there were high percentages of people who always smoked, women, and patients with adenocarcinoma, which may be a clinical feature of lung cancer patients in China, or it may be the cause of a unique subgroup of cases.

## Discussion

To our knowledge, this is the most comprehensive report to evaluate associations of lymphocyte subsets in relation to the presence of cancer occurrence and lung cancer stage.

We discovered that levels of NK cells, CD4+ T cells, naïve CD4+/CD4+, naïve CD4+ T cells, CD4+CD28+ T cells were significantly different in lung cancer patients versus healthy individuals and that the percentages of the different cell subsets are associated with lung cancer stage.

Several reports have demonstrated the predictive role of lymphocyte subsets in cancers, however, those results are controversial and not comprehensive. We evaluated the predictive role of lymphocyte subsets in carcinogenesis. In this study, we found that lymphocyte subsets were associated with cancer occurrence and lung cancer stage, which is consistent with other previously published studies articles [12–14]. However, conflicting results have also been reported in several studies, such as high CD8+ T cells and decreased CD4+ T cell counts, and CD4+/CD8+ ratio in patients with NSCLC than those in controls [15]. CD8+ T cells and CD4+ T cells undergo a period of massive expansion, activation, differentiation into effector cells, and apoptosis, which might lead to these disparate results. As the cytotoxicity cells, low NK cell counts and CD8+ T cell counts might imply that weakened immunological system contributes to growth of cancer cells by effectively reducing the killing effect toward the cancer cells.. As the helper cells, decreased naïve CD4+/CD4+ percentages and increased CD4+ T cell counts, memory CD4+/CD4+ percentages might suggest that the anti-tumor immune response was activated and naïve CD4+ T cells were differentiated into CD4+ T cells and memory CD4+ T cells [16]. CD28 are a very important co-stimulatory marker, which is required as a secondary signal for activated CD8+ T cells and CD4+ T cells exerting anti-tumor response. We discovered patients had higher CD4+CD28+/CD4+ percentage and CD4+CD28+ T cell counts than those in controls, which might imply that CD4+ T cells were activated in cancer occurrence. Noteworthily, patients had high CD8+CD28+/CD8+ percentage than that in controls, but there was no significant difference in the counts of CD8+CD28+ between patients and controls. These results might imply that the activation CD8+ T cells was limited, as a result cancer occurrence based on the reduced antitumor. HLA-DR and CD38, as markers of CD8+ T cell activation, play a crucial predictive value in CD8+ T cells activation and CD4+ T cells depletion [17]. Elevated levels of HLA-DR and CD38 have suggested the immune system was activated during tumorigenesis. The CD4+/CD8+ ratio is a marker of cell-mediated immunity in cancer patients [18]. Decreased ratio is reported to link with a low immunological function [19].

Immune status is closely associated with the pathogenesis and development of cancer. Less research has been reported on the role of peripheral blood immune cells in advance cancer stage, which focus on B cells, NK cells, CD4+ T cells, CD8+ T cells and CD4+/CD8+ [12, 14]. In addition, there is no consensus regarding change of lymphocyte subsets in the advance cancer stage. Liang et al. reported that there was decreased trend in counts of NK cells, CD4+ T cells and CD4+/CD8+ ratio with advanced NSCLC (including stage III, IV and controls groups), and no relationship between CD8+ T cells and stage [14]. Mazzoccoli et al. reported that there were decreasing trend for B cells and increasing trend for NK cells in cancer stage [12]. Those results were not exactly the same as ours. In our study, advance cancer stage was negatively associated with levels of B cells, CD4+ T cells, naïve CD4+/CD4+, naïve CD4+ T cells, CD4+CD28+ T cells, CD8+CD28+ T cells and positively associated with NK cells, WBC counts, monocytes, neutrophils, eosinophils, basophils, MLR, NLR, ELR, BLR, PLR. A possible explanation for this finding could be immune function disorder associated with clinical staging. B cells can recognize antigens, regulate process and presentation of antigen, present antigens, provide co-stimulation [20]. As to our results of lymphocyte subsets might suggest that immune function is severely damaged with advancing stage causing growth and metastasis of cancer cells. The reason is likely that decreased expression of co-stimulatory molecule (CD28) can suppress anti-tumor response by limiting aggregation of CD4+ T cells, CD8+ T cells and immune system were not activated during disease progression due to no significant difference for HLA and CD38 in each stage. WBC and neutrophils can contrubite to disease progression and metastasis, which could reflect the tumor burden in patients [21]. Increased neutrophil levels might inhibit the antitumor effects of T cells and NK cells Increased NLR levels represents increased inflammation and decreased immune reaction [22]. Several reports have been demonstated that the change of WBC, monocytes, neutrophils, eosinophils, basophils, MLR, NLR, ELR, BLR, PLR were associated with cancer prognosis in some solid tumors [23, 24]. However, there is no reported for the predictive value of those cells in cancer occurrence and progression. In this study, results about elevated levels of inflammatory cells might demonstrate that those cells play an important role in anti-tumor response and can predict cancer progression not just prognosis. In short, those immune cells are gradually destroyed with advancing cancer, which restricts the recognition and killing of cancer cells and triggers the extensive dissemination of cancer cells.

There are several limitations in this study. First, threshold value had not been provided in this work which needs further investigations. Second, limited numbers of patients with stage II and III and in homogenous clinicopathologic characteristics of samples. Last, this paper lacks a validation queue, and we will continue to collect samples to further verify the results. Despite these limitations, this study demonstrated that immune cells play a predictive role in the NSCLC development and progression.

In summary, our findings show a significant relationship between lymphocyte subset/myeloid cells in the presence of lung cancer compared to healthy individuals and significant relationship to these immune parameters and lung cancer stage. Those results of our study may suggest potential strategies for screening, prevention or treatment of lung cancer.

## Supplementary Information

**Additional file 1. Table S1**: Relationship between immune cells levels and basic parameters for NSCLC patients.

## Data Availability

The datasets used and/or analyzed during the current study are available from the corresponding author upon reasonable request.

## Abbreviations

NSCLC: Non-small cell lung cancer
WBC: White blood cells
MLR: Monocytes to lymphocyte ratio
NLR: Neutrophils to lymphocyte ratio
ELR: Eosnophil to lymphocyte ratio
BLR: Basophil to lymphocyte ratio
PUMCH: Peking Union Medical College Hospital
RBC: Red blood cells
RLR: Red blood cells to lymphocyte ratio
HLR: Hemoglobin to lymphocyte ratio
