# Supplementary material for: Circulating activated immune cells as a potential blood biomarkers of non-small cell lung cancer occurrence and progression
Source: BMC Pulm Med. 2021 Sep 6;21:282. doi: 10.1186/s12890-021-01636-x (PMC8420051; doi:10.1186/s12890-021-01636-x)
Supplement: Supplementary file 1 — Additional file 1. Table S1: Relationship between immune cells levels and basic parameters for NSCLC patients. [file 12890_2021_1636_MOESM1_ESM.docx]

Supplementary Table 1 Relationship between immune cells levels and basic parameters for NSCLC patients

| Immune cells | Stage I | Stage II | Stage III | Stage IV | r | *p* |
| --- | --- | --- | --- | --- | --- | --- |
| T lymphocytes | 1279.00 ± 447.40 | 1431.89 ± 591.01 | 1204.15 ± 460.24 | 1141.48 ± 396.68 | -0.094 | 0.109 |
| B cells (cells/ul) | 196.98 ± 97.43 | 192.50 ± 93.05 | 166.51 ± 90.52 | 145.72 ± 90.52 | **-0.193** | **0.001** |
| NK cells (cells/ul) | 248.97 ± 188.21 | 364.00 ± 246.53 | 213.51 ± 120.56 | 330.67 ± 302.36 | **0.117** | **0.045** |
| CD4+ T cells (cells/ul) | 767.86 ± 302.76 | 860.72 ± 328.86 | 678.32 ± 313.60 | 671.32 ± 279.37 | **-0.135** | **0.020** |
| CD8+ T cells (cells/ul) | 447.11 ± 206.18 | 460.48 ± 249.63 | 447.24 ± 257.66 | 397.70 ± 198.3 | -0.085 | 0.148 |
| Memory CD4+/ CD4+ (%) | 72.46 ± 13.19 | 76.71 ± 10.88 | 75.00 ± 10.83 | 75.95 ± 12.17 | 0.106 | 0.070 |
| Memory CD4+ T cells (cells/ul) | 545.61 ± 206.45 | 655.60 ± 265.61 | 508.79 ± 252.94 | 507.09 ± 242.88 | -0.086 | 0.141 |
| Naïve CD4+/CD4+ (%) | 25.31 ± 12.33 | 21.22 ± 10.93 | 22.32 ± 10.58 | 21.89 ± 12.04 | **-0.122** | **0.037** |
| Naïve CD4+ T cells (cells/ul) | 196.67 ± 134.54 | 186.14 ± 114.84 | 153.02 ± 104.00 | 149.67 ± 101.18 | **-0.144*** | **0.013** |
| CD4+CD28+/CD4+ (%) | 93.17 ±  7.53 | 92.13 ±  8.09 | 93.39 ± 10.04 | 88.73 ± 13.12 | -0.082 | 0.163 |
| CD4+CD28+ T cells (cells/ul) | 696.05 ± 268.94 | 787.32 ± 302.75 | 640.89 ± 317.51 | 583.34 ± 219.81 | **-0.137** | **0.019** |
| CD8+CD28+/CD8+ (%) | 56.88 ± 16.46 | 58.34 ±  19.10 | 53.78 ±  18.70 | 52.20 ±  18.79 | -0.089 | 0.129 |
| CD8+CD28+T cell (cells/ul) | 243.01 ± 109.03 | 243.44 ± 109.52 | 216.34 ± 114.09 | 186.20 ±  81.8 | **-0.186** | **0.001** |
| CD8+HLA-DR/CD8+ (%) | 37.05 ±  13.24 | 40.59 ±  17.46 | 39.43 ±  16.11 | 40.76 ±  12.99 | 0.112 | 0.055 |
| CD8+HLA-DR T cells (cells/ul) | 169.76 ± 119.33 | 205.78 ± 163.59 | 191.29 ± 181.21 | 168.68 ± 109.18 | 0.003 | 0.958 |
| CD8+CD38+/CD8+ (%) | 29.53 ±  11.56 | 31.29 ±  15.61 | 31.58 ±  15.39 | 35.05 ±  15.59 | 0.091 | 0.119 |
| CD8+CD38+ T cells (cells/ul) | 130.97 ± 79.15 | 142.87 ± 113.22 | 148.71 ± 144.44 | 146.02 ± 121.53 | -0.026 | 0.651 |
| CD4+/CD8+ (%) | 1.94 ± 0.91 | 2.16 ± 0.85 | 1.94 ± 1.09 | 2.12 ± 1.40 | 0.014 | 0.816 |
| WBC (cells/10^12ul) | 6.71 ±  3.23 | 6.36 ±  2.10 | 6.70 ±  2.27 | 7.13 ±  2.10 | **0.177** | **0.002** |
| Lymphocytes (cells/10^12ul) | 1.77 ±  0.60 | 2.01 ±  0.71 | 1.60 ±  0.55 | 1.65 ±  0.53 | -0.058 | 0.326 |
| Monocytes (cells/ul) | 0.36 ±  0.16 | 0.41 ±  0.16 | 0.42 ±  0.22 | 0.42 ±  0.15 | **0.186** | **0.001** |
| Neutrophils (cells/ul) | 4.47 ±  3.26 | 3.70 ±  1.51 | 4.30 ±  2.29 | 4.80 ±  1.97 | **0.158** | **0.007** |
| Eosnophils (cells/ul) | 0.12 ±  0.11 | 0.13 ±  0.08 | 0.15 ±  0.21 | 0.20 ±  0.21 | **0.171** | **0.003** |
| Basophils (cells/ul) | 0.03 ±  0.01 | 0.03 ±  0.02 | 0.03 ± 0.02 | 0.04 ±  0.02 | **0.203** | **<0.001** |
| RBC (cells/ul) | 4.51 ±  0.43 | 4.31 ±  1.16 | 4.53 ±  0.39 | 4.69 ±  0.56 | 0.092 | 0.114 |
| Hemoglobins (cells/ul) | 138.63 ± 12.48 | 136.56 ± 17.12 | 133.89 ± 15.53 | 140.78 ± 18.37 | 0.005 | 0.929 |
| Platelets (cells/ul) | 221.51 ± 50.71 | 229.56 ± 70.55 | 228.70 ± 80.14 | 255.59 ± 88.44 | 0.113 | 0.053 |
| MLR | 0.23 ± 0.15 | 0.22 ± 0.08 | 0.29 ± 0.18 | 0.29 ± 0.17 | **0.206** | **<0.001** |
| NLR | 2.95 ± 2.87 | 1.97 ± 0.86 | 3.03 ± 2.07 | 3.51 ± 3.03 | **0.165** | **0.005** |
| ELR | 0.07 ± 0.06 | 0.07 ± 0.04 | 0.11 ± 0.15 | 0.24 ± 0.73 | **0.188** | **0.001** |
| BLR | 0.02 ± 0.01 | 0.02 ± 0.01 | 0.02 ± 0.01 | 0.06 ± 0.23 | **0.230** | **<0.001** |
| RLR | 2.90 ± 1.19 | 2.41 ± 1.07 | 3.17 ± 1.28 | 3.26 ± 1.73 | 0.068 | 0.244 |
| HLR | 89.11 ± 36.46 | 75.10 ± 26.05 | 93.62 ± 39.23 | 98.76 ± 54.84 | 0.037 | 0.525 |
| RLR | 139.46 ± 56.65 | 125.64 ± 52.47 | 156.00 ± 66.78 | 170.25 ± 86.91 | **0.121** | **0.038** |
